# Influence of Temperature and Polymer Concentration on the Nonlinear Response of Highly Acetylated Chitosan–Genipin Hydrogels

**DOI:** 10.3390/gels8030194

**Published:** 2022-03-21

**Authors:** Lorenzo Mio, Pasquale Sacco, Ivan Donati

**Affiliations:** 1 Department of Life Sciences, University of Trieste, Via Licio Giorgieri 5, I-34127 Trieste, Italy; lorenzo.mio@studenti.units.it (L.M.); psacco@units.it (P.S.); 2 AREA Science Park, Loc. Padriciano 99, I-34149 Trieste, Italy; 3 Department of Medicine, Surgery and Health Sciences, University of Trieste, Piazza dell’Ospitale 1, I-34129 Trieste, Italy

**Keywords:** hydrogel, chitosan, genipin, strain hardening, neutral pH

## Abstract

Strain hardening, i.e., the nonlinear elastic response of materials under load, is a physiological response of biological tissues to mechanical stimulation. It has recently been shown to play a central role in regulating cell fate. In this paper, we investigate the effect of temperature and polymer concentrations on the strain hardening of covalent hydrogels composed of pH-neutral soluble chitosans crosslinked with genipin. A series of highly acetylated chitosans with a fraction of acetylated units, F_A_, in the range of 0.4–0.6 was synthesized by the homogeneous re-N-acetylation of a partially acetylated chitosan or the heterogeneous deacetylation of chitin. A chitosan sample with an F_A_ = 0.44 was used to prepare hydrogels with genipin as a crosslinker at a neutral pH. Time and frequency sweep experiments were then performed to obtain information on the gelling kinetics and mechanical response of the resulting hydrogels under small amplitude oscillatory shear. While the shear modulus depends on the chitosan concentration and is almost independent of the gel temperature, we show that the extent of hardening can be modulated when the gelling temperature is varied and is almost independent of the experimental conditions used to build the hydrogels (ex situ or in situ gelation). The overall effect is attributed to a subtle balance between the physical (weak) entanglements and covalent (strong) crosslinks that determine the mechanical response of highly acetylated chitosan hydrogels at large deformations.

## 1. Introduction

Chitosan is a binary heteropolysaccharide consisting of β-1→4-linked 2-acetamido-2-deoxy-D-glucopyranose (N-acetyl-glucosamine, A unit) and 2-amino-2-deoxy-D-glucopyranose (glucosamine, D unit) in varying proportions and patterns along the polymer chain. Chitosan is produced via the deacetylation of chitin, a structural component of the exoskeleton of Arthropoda and of the cell walls of certain fungi such as Zygomycetes [1,2]. The water solubility of the polysaccharide is modulated by the proportion of the remaining N-acetyl-glucosamine units. While in principle all chitosans are soluble under acidic conditions, they are only soluble at neutral pH if the fraction of acetylated units, F_A_, is between 0.4 and 0.6 [3].

Chitosan is a polysaccharide with antibacterial activity, and is nontoxic, nonimmunogenic and biodegradable [4]. Chitosan-based systems, such as microspheres, nanoparticles, films and hydrogels, are also very interesting for biotechnology and biomedicine [5,6,7,8,9,10]. Several ways for producing both physical and chemical [11] chitosan hydrogels have been published in the literature. Aldehydes, epoxy compounds, esters [12,13,14,15], tetrakis-(hydroxymethyl)-phosphonium chloride (THPC) [16] and acrylate moieties were used as chemical reticulating agents [17].

Genipin is a chemical crosslinker that has recently attracted attention [18]. The hydrolysis of geniposide isolated from the fruits of Gardenia jasminoides Ellis produces the monoterpenoid genipin (methyl 1-hydroxy-7-(hydroxymethyl)-1,4a,5,7a-tetrahydrocyclopenta [c]pyran-4-carboxylate). Compared to other covalent crosslinkers, genipin is significantly less toxic [19]. Genipin has been investigated as a chitosan crosslinking agent under acidic conditions for the preparation of tissue engineering hydrogels [20,21]. However, there are few examples of genipin–chitosan hydrogels prepared at a neutral pH. Recently, our research group has shown that highly acetylated chitosan samples are suitable for this purpose and that the hydrogels prepared exhibit a strain-hardening effect [22].

When biological tissues are mechanically stimulated, strain hardening, defined as an increase in the elastic response under stress or strain, is a typical phenomenon. It has been postulated that strain hardening protects tissues from rupture under high stress, but it may also play a role in tissue development, homeostasis and repair [23].

The rheological behavior of the extracellular matrix (ECM) has been shown to regulate cell shape and function, and alterations may contribute to the development of diseases [24]. Arterial stiffening, for example, has been linked to cardiovascular and renal outcomes in diabetics [25]. The native ECM is a complex matrix with pronounced viscoelasticity and extremely dynamic mechanical properties. The time-dependent response of the native ECM to the action of stress is crucial for its interactions with cells. Indeed, the tensile forces of cells are used to remodel and reorganize the matrix to achieve spreading, proliferation and migration [26].

In addition, the natural ECM exhibits nonlinear elasticity and strain hardening, which in some situations occurs even at extremely low loads [27]. The nonlinear elasticity of the ECM has been shown to allow cells to respond to mechanical signals emitted by other, more distant cells [28]. To approximate the biomechanical properties of the ECM that determine cell behavior and fate, as well as disease progression, the nonlinear, load-dependent and time-dependent mechanical outcomes of the materials need to be investigated. It is becoming increasingly clear that it is not sufficient to match the elastic modulus of tissues with hydrogels to create a suitable matrix for tissue regeneration. Nonlinear reactions as well as the time-dependent reactions should be taken into account [23].

Synthetic polymers rarely meet the requirements for strain hardening, but biological polymers such as collagen, fibrin and actin do. The strain fields of collagen gels can be very nonuniform, whereas the strain fields of polyacrylamide gels decrease uniformly [23]. Strain hardening in biopolymer-based hydrogels has been attributed to differences in the microstructural arrangement or topological properties [29]. The efficient tuning of the nonlinear properties of a hydrogel, especially the strain-hardening effect, would be beneficial to study cell fate and attachment in detail, and to develop new bioactive biomaterials.

In the present work, highly acetylated chitosan hydrogels crosslinked with genipin were prepared, and their nonlinear properties, specifically strain hardening, were investigated by varying the experimental conditions.

## 2. Results and Discussion

### 2.1. Synthesis of Acetylated Chitosans

The solubility of chitosan at a neutral pH, which is achieved with an F_A_ in the range 0.4–0.6, is crucial for the preparation of genipin-based hydrogels. For the synthesis of highly acetylated chitosans, we used two alternative methods: the homogeneous re-N-acetylation of a chitosan sample with a low F_A_ and the heterogeneous deacetylation of chitin. Table 1 lists the acetylated chitosan samples produced in the present study.

Under homogeneous conditions, the reaction with acetic anhydride as an acetyl group donor resulted in an almost random reacetylation of the chitosan [9,30] to F_A_ = 0.44. With almost no loss of molecular weight, an efficiency of the reaction of 89% was observed. Heterogeneous chitin deacetylation, which has been shown to lead to a more blockwise arrangement of GlcNAc sequences [31], is also effective. The remaining degree of acetylation after a 30 min treatment with concentrated sodium hydroxide was 54%, which is quite close to the solubility limit. Extending the treatment time to 60 min resulted in a lower degree of residual acetylation, but at the cost of increased chain scission and consequently a decrease in molecular weight. Overall, ACh1 proved to be a good compromise for the preparation of genipin-based hydrogels, with a sufficient degree of acetylation and a high molecular weight. This sample was used for the preparation of hydrogels.

### 2.2. Genipin-Based Hydrogels from Highly Acetylated Chitosan

**Figure 1:**
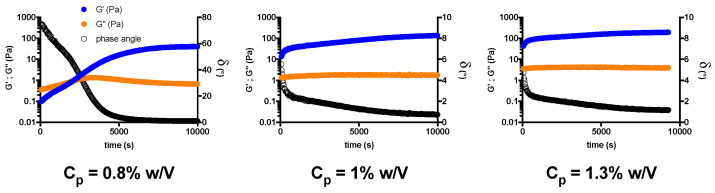
Gelling kinetics of highly acetylated chitosan sample (ACh1). Dependence of storage modulus (*G*′, in blue), viscous modulus (*G*″, in orange) and phase angle (empty circles) as a function of time. Final experimental conditions: (ACh1) = 0.8–1.3% w/V, (genipin) = 0.31 mM, gelling temperature 60 °C, 1× PBS as solvent, pH 7.4.

Following a previously described technique, sample ACh1 was used to prepare genipin-based hydrogels [22]. The gelation kinetics were monitored in the rheometer for three hours. Different hydrogels were prepared by changing the final concentration of chitosan while keeping the amount of genipin constant. As a result, the glucosamine-to-genipin molar ratio (R_D/G_) of the different hydrogels varied, significantly affecting the kinetics of the hydrogel formation. At a chitosan concentration of 0.8%, the system initially behaved more like a viscous than an elastic material, with *G*″ > *G*′ occurring for approximately the first 2000 s, followed by a crossover of moduli and a more solid-like behavior. When the chitosan concentration was increased to 1%, the system exhibited a storage modulus greater than the viscous modulus from the first time points, and *G*′ continued to increase with time. Finally, when the chitosan concentration was increased to 1.3%, *G*′ showed a further modest increase with time in addition to the elastic behavior from the first time point (Figure 1).

Following the time sweep experiments, the shear modulus (*G*) of the hydrogels was calculated using frequency sweep tests interpreted in terms of the generalized Maxwell model, consisting of a set of parallel elements (spring and dashpot) to which an additional spring was added [32,33] (see Supplementary Materials). The following equations (Equations (1) and (2)) can be used to model the storage and loss moduli as a function of pulsation, ω:

(1) $$G^{\prime }=G_{e}+\sum_{i=1}^{N}G_{i}\frac{{\left(\lambda_{i}\omega \right)}^{2}}{1+{\left(\lambda_{i}\omega \right)}^{2}},$$

(2) $$G^{\prime \prime }=\sum_{i=1}^{N}G_{i}\frac{\lambda_{i}\omega }{1+{\left(\lambda_{i}\omega \right)}^{2}},$$

with

$$G_{i}=\frac{\eta_{i}}{\lambda_{i}}$$

where *N* is the number of Maxwell elements considered and *G_i_*, *η_i_* and *λ_i_* represent the spring constant, the dashpot viscosity and the relaxation time of the *i*th Maxwell element, respectively. *G_e_* is the spring constant of the last Maxwell element which is supposed to be purely elastic [33]. The fitting of the experimental data was performed assuming that the relaxation times are not independent each other but they are scaled by a factor 10. Therefore, the parameters of the model are *G_e_*, *η_i_* and *λ_i_*. The number of Maxwell elements was selected, based on a statistical procedure, to minimize the product *χ*^2^∙*Np*, where *χ*^2^ is the sum of the squared errors, while *Np* (=2 + *N*) indicates the number of fitting parameters. In all cases analyzed, the experimental results were best fitted using three parallel elements.

By using the generalized Maxwell model, the shear modulus can be calculated as (Equation (3)):

(3) $$G=G_{e}+\sum_{i=1}^{N}G_{i},$$

**Figure 2:**
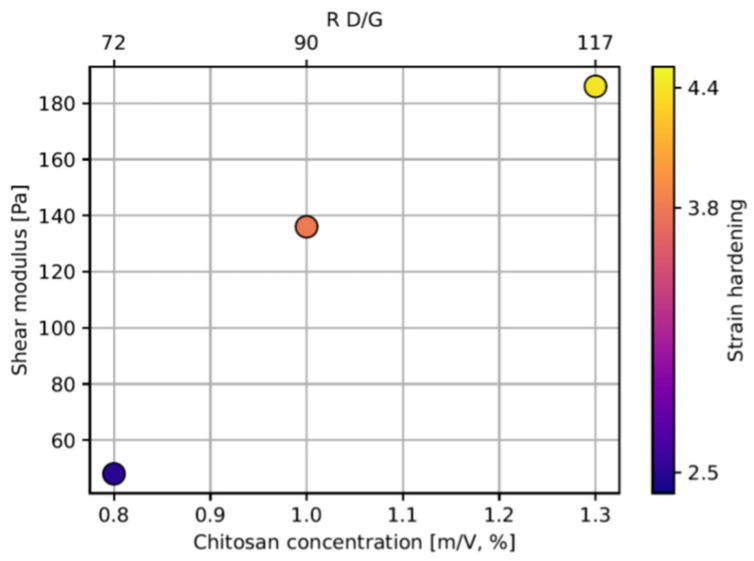
Dependence of the shear modulus from chitosan concentration for genipin-based hydrogels. The color bar on the right-end side represents the strain hardening of the hydrogels, defined as ${G^{\prime }}_{max}/{G^{\prime }}_{0}$ (see Equation (4)). The amount of genipin used for all hydrogels was 0.31 mM. The value of R_D/G_ associated with each hydrogel is reported on the upper *x*-axis. Chitosan concentration is varied in the range from 0.8% to 1.3%.

The shear modulus of the hydrogels rose with the increasing chitosan concentration at a constant amount of genipin (Figure 2). When a larger number of available amino groups were used, more crosslinks were formed. Increasing the chitosan concentration from 0.8% to 1.3% resulted in a 3.9-fold increase in *G*. While the amount of genipin remained constant, the strain hardening increased with the amount of chitosan.

Next, stress sweep experiments were undertaken to evaluate the mechanical response of the hydrogels at large deformations. Interestingly, the magnitude of strain hardening, calculated as ${G^{\prime }}_{max}/{G^{\prime }}_{0}$ (see Equation (4)), scaled with the chitosan concentration (Figure 2). This behavior can be safely ascribed to the formation of more physical entanglements throughout the network due to the higher flexibility of the polymer chains, i.e., an increased R_D/G_ [22].

**Figure 3:**
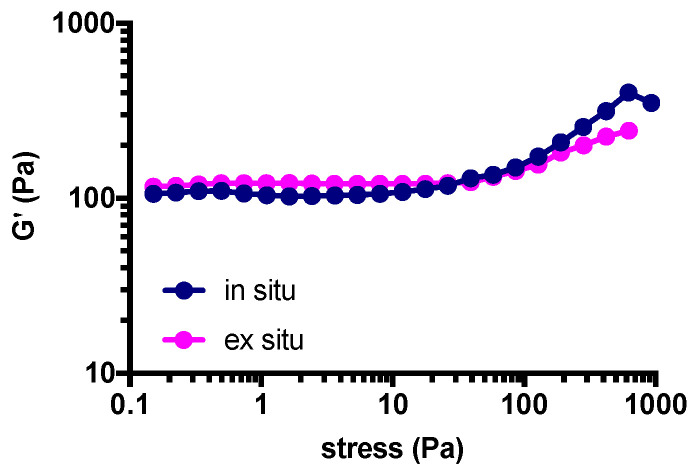
Sample-case dependence of the storage modulus (*G*′) as a function of stress for genipin-based chitosan hydrogels prepared on the rheometer (in situ, blue dots) or on plastic wells (ex situ, purple dots). In both cases, the polymer concentration was 1% (m/V), the genipin concentration was 0.31 mM, T = 60 °C and setting time 3 h.

Curiously, a similar mechanical performance was found when the genipin-based chitosan hydrogels with ACh1 = 1% *w*/*V* and R_D/G_ = 90 were incubated in plastic wells (ex situ gelation) in the same experimental conditions (3 h, T = 60 °C) (Figure 3). The linear elasticity showed no differences with respect to in situ (i.e., on rheometer) gelation. At the same time, the stiffening effect is present but to a slightly lesser extent, which is probably due to partial sliding phenomena associated with the different experimental set-up. It can be concluded that the experimental setting of the hydrogel production has a minor effect on the overall properties of the final construct, in contrast to what has been found for agarose-based networks [34].

### 2.3. Effect of Gelling Temperature on the Strain-Hardening Effect

**Figure 4:**
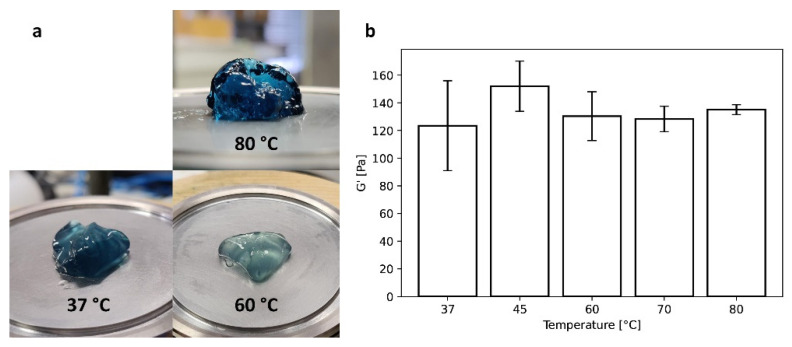
Effects of gelling temperature on chitosan–genipin-based hydrogels (polymer concentration = 1%, RD/G = 90): (**a**) photographs of the genipin-based hydrogels obtained at different temperatures; (**b**) dependence of the storage modulus (*G*′) recorded at 1 Pa from temperature for genipin-based chitosan hydrogels. No significant difference was found in the storage modulus of the different gels.

Genipin-based hydrogels were prepared with a 1% concentration of ACh1 chitosan at a different temperature but with the same R_D/G_ of 90. Although the starting solutions were chemically identical, the hydrogels differed significantly in color, with the intensity of the blue depending on the temperature used: a dark blue was obtained at both 80 °C and 37 °C, while the gels at 60 °C were characterized by a lighter blue (Figure 4a). In the presence of oxygen, the dimerization of genipin molecules attached to amino groups leads to the formation of dark blue colored adducts [18]. However, a complicated pattern of different reactions between genipin molecules in the presence of oxygen has been described in the literature [35]. The hydrogels were synthesized in the presence of oxygen.

The mechanical performance of the hydrogels synthesized at temperatures ranging from 37 to 80 °C was measured. Figure 4b shows that the shear modulus was largely unaffected by the temperature, which means that the number of elastically active chains in the hydrogel remained the same. The temperature therefore has an influence on the kinetics of the reaction between genipin and chitosan [36], but not on the overall mechanical properties. Given the difference in color, it can also be assumed that the temperature causes different interactions between the genipin amounts in the different samples.

**Figure 5:**
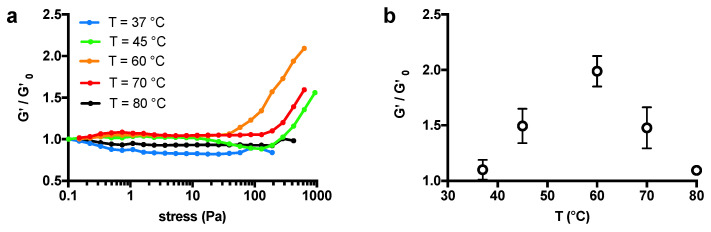
Effect of the temperature on the nonlinear behavior of chitosan–genipin hydrogels. (**a**) Dependence of the ratio ${G^{\prime }}_{max}/{G^{\prime }}_{0}$ from stress for genipin-based chitosan hydrogels formed at 37 °C (blue), 45 °C (light green), 60 °C (orange), 70 °C (red) and 80 °C (black), respectively. (**b**) Highest value of ${G^{\prime }}_{max}/{G^{\prime }}_{0}$ reached at different temperatures. Conditions: polymer concentration = 1% (m/V), glucosamine-to-genipin molar ratio (R_D/G_) = 90.

The hydrogels developed at different temperatures have different properties in the nonlinear region, i.e., at high loads, but have a similar mechanical performance at the low-load region (Figure 5a). The Soskey–Winter equation (Equation (4)) [37,38] was used to fit the experimental data into the linear response range (see Supplementary Materials).

(4) $$G^{\prime }=\frac{{G^{\prime }}_{0}}{1+{\left(b\gamma \right)}^{n}}$$

where ${G^{\prime }}_{0}$ is the limiting values of the storage modulus for $\gamma_{\to 0}$, while *b* and *n* are adjustable parameters. Since the fit is performed on the linear *G*′-γ region, in all cases *b* = 0.

A strain-hardening effect was observed in the hydrogels prepared at 45, 60 and 70 °C, respectively, but not in the hydrogels prepared at 37 °C and 80 °C, respectively. The nonmonotonic trend of ${G^{\prime }}_{max}/{G^{\prime }}_{0}$ with the temperature is shown in Figure 5b, with a maximum at a temperature of 60 °C. Overall, the temperature regulates strain hardening.

**Scheme 1:**
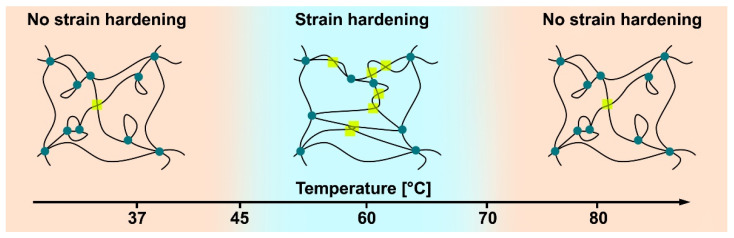
Proposed mechanism for the effect of temperature on strain hardening of highly acetylated chitosan–genipin hydrogels. The gel reticulum is schematically represented in three different conditions. In all of those, genipin crosslinks are represented as blue dots, while physical entanglements are indicated with green squares. The establishment of physical entanglements between chitosan chains is associated with strain hardening.

The hydrogels prepared at different temperatures show a similar density of polymer chains contributing to the mechanical properties at low strain (i.e., elastically active chains) (Figure 4b). Differences arise upon increasing the stress applied to the sample. Strain hardening was described as an imbalance of entanglement versus disentanglement between semiflexible chitosan chains. Entanglements between the polysaccharide chains are described as temporary crosslinks which are formed when the critical strain is exceeded, leading to strain hardening. This model was developed by incubating the hydrogel at 60 °C [22]. Based on the results of this study, strain hardening could be the result of an equilibrium between two phenomena: (i) the probability of temporary entanglement between the polymer chains in the forming gel; (ii) the reaction rate of genipin. Three situations are possible. At temperatures lower than 45 °C, polymer chains diffuse slowly, and genipin is more likely to permanently crosslink different segments of the same chain, replacing the entanglements and so preventing their formation. At temperatures between 45 °C and 70 °C, polymer chains diffuse more quickly, interacting with each and resulting in temporary entanglements together with genipin crosslinks. At temperatures higher than 70 °C, both the kinetics of the genipin reaction and the polymer diffusion are very fast. This likely leads to crosslinks within the same elastically active chain, reducing the probability of entanglements (Scheme 1).

**Figure 6:**
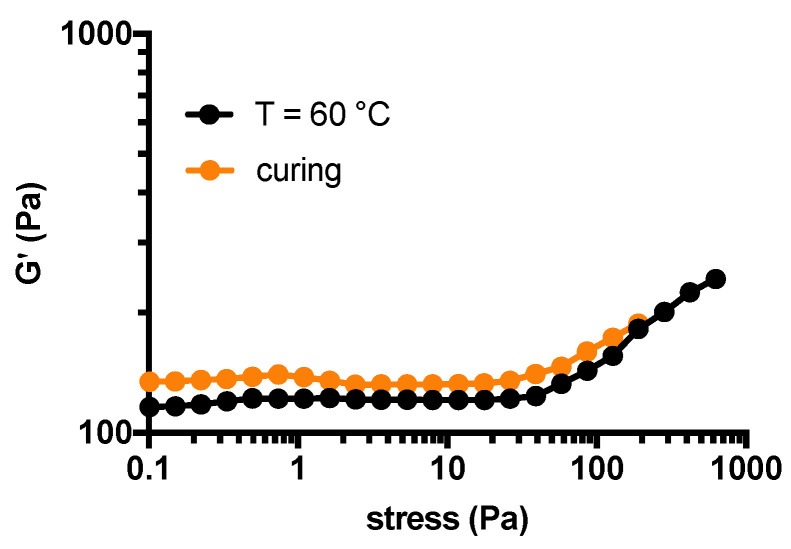
Dependence of storage modulus (*G*′) as a function of stress for genipin-based chitosan hydrogel from ACh1 cast at 60 °C (black) and after a curing at 37 °C for 24 h (orange).

The hydrogel produced at 60 °C is dynamic in its state. After casting the hydrogel at 60 °C, the system was kept at 37 °C for 24 h, resulting in a modest increase in the storage modulus and consequently a decrease in strain hardening. This could be due to an increase in the crosslinks between the elastically active chains, which prevent entanglements from forming as the stress increases (Figure 6).

## 3. Conclusions

In this study, we investigated the strain hardening of chitosan hydrogels prepared by the covalent crosslinking of genipin. To achieve this goal, we first synthesized pH-neutral soluble chitosans that have a degree of acetylation in the range of 40–60%. This was done either by the standard homogeneous re-N-acetylation of a partially acetylated chitosan template using acetic anhydride as an acetyl group donor or by the heterogeneous deacetylation of chitin. While the latter process led to more or less strong chain scission due to the strong alkaline conditions used in the experimental procedure, homogeneous acetylation led to mild experimental conditions with negligible depolymerization. These chitosans are soluble at a neutral pH and were used to form the covalent hydrogels in the presence of genipin as a crosslinker. We have found that the gelling temperature is a crucial experimental parameter that must be taken into account to modulate the mechanical response at large deformations. Indeed, we have shown here a nonmonotonic trend of the extent of the strain hardening as a function of the gelling temperature. Interestingly, although the nonlinear elasticity varies with the temperature, the elastic response does not, and the experimental procedure used to prepare the hydrogels does not affect their final performance at large deformations. Overall, the results presented in this work provide insight into the nonlinear elasticity of the chitosan–genipin gelling system and could be the basis for the development of biomaterials with tunable mechanical properties useful in the biomedical field.

## 4. Materials and Methods

### 4.1. Materials

NovaMatrix/FMC Biopolymer (Sandvika, Norway) kindly provided the high molecular weight chitosan (HMWc) with an acetylated unit content, FA, of 0.14 in the base form (GlcNH_2_). The intrinsic viscosity, [*η*], was determined to be 988 mL/g and the viscosity average molecular weight determined to be 320,000 by capillary viscometry. Chitin from crab shells, phosphate buffered saline (PBS), deuterium oxide (D_2_O), deuterium chloride (DCl), sodium deuterium oxide (NaOD), sodium nitrite (NaNO_2_) and sodium hydroxide (NaOH) were all purchased from Sigma-Aldrich (St. Louis, MO, USA) The composition of PBS was 137 mM NaCl, 2.7 mM KCl and 10 mM phosphate buffer, with an ionic strength (I) of ~168 mM and pH of 7.4. Genipin was purchased from Challenge Bioproducts Co., Ltd. (Taiwan). All reagents and chemicals were of high purity. Deionized water was used for all preparations. Methodologies used are similar to those in [22].

### 4.2. Homogeneous Acetylation of Chitosan

HMWc (4 g) was dispersed in water (400 mL) and glacial acetic acid (4 mL) was added for solubilization of the polysaccharide. The solution was stirred for 2 h at room temperature, then ethanol (400 mL) was added, and the solution was stirred for another 2 h. Acetic anhydride (800 μL) was added dropwise at room temperature and the resulting solution was stirred overnight. The acetylated chitosan was precipitated by dropwise addition of a mixture of 70% ethanol and 30% isopropyl alcohol (ETA/IPA) in the presence of NaCl with stirring. The supernatant of the suspension was removed, and the precipitate was washed four times with mixtures of ETA/IPA and decreasing amounts of water. The precipitate was washed twice more with ETA–IPA, the powder was filtered through a sintered glass filter and vacuum-dried.

### 4.3. Heterogeneous Deacetylation of Chitin

Chitin flakes (10 g), milled to a particle size lower than 1 mm, were added to ice-cold 20 M aqueous NaOH (172 mL). Chitin was allowed to swell at 10 °C for 3 days under magnetic stirring. The suspension was kept at 60 °C under magnetic stirring for 30 min or 1 h to obtain a different degree of final acetylation. The deacetylated chitin was extensively washed with warm deionized water and collected by centrifugation (1000× *g*, 5 min). The purification procedure was repeated eight times. The solid was dried overnight at room temperature and extensively washed with deionized warm water until a neutral pH was reached. Acetylated chitosan was further dried at room temperature, and then converted into its hydrochloride form suspending the product in deionized water, to which was added HCl 1 M, to a final pH~4. The acetylated chitosan in hydrochloride form was filtered through Millipore filters (∅ 3 μm) and freeze-dried.

### 4.4. Viscosity Measurements

Intrinsic viscosity [*η*] was measured at 25 °C by means of a Schott-Geräte AVS370 automatic measuring apparatus, equipped with a CT 72/P thermostat (SI Analytics), using an Ubbelohde-type capillary viscometer. Intrinsic viscosity values were determined by analyzing the polymer concentration dependence of the reduced specific viscosity (*η_sp_/c*) and of the reduced logarithm of the relative viscosity (*ln(η_rel_)/c*) using the Huggins (Equation (5)) and Kraemer (Equation (6)) equations, respectively:

(5) $$\frac{\eta_{sp}}{c}=\left[\eta \right]+k_{H}{\left[\eta \right]}^{2}c,$$

(6) $$\frac{ln\eta_{rel}}{c}=\left[\eta \right]-k_{K}{\left[\eta \right]}^{2}c,$$

A buffer solution composed of 20 mM AcOH/AcNa (pH 4.5) and 100 mM NaCl was used as solvent [9].

### 4.5. ^1^H-NMR Spectroscopy

Chitosan samples were prepared as follows: 20 mg of the polymer was dissolved in 2 mL of deuterium oxide, and 150 μL of 1 M DCl was added with vigorous stirring and gentle heating. Then, 30 μL of aqueous NaNO_2_ (10 mg/mL) was added, and the resulting solution was stirred for 2 h. The pD was increased to 3–4 with a solution of NaOD, and 700 μL of the chitosan samples were transferred to NMR tubes and analyzed using a Varian MR-400 NMR spectrometer working at 400 MHz. The spectra were recorded at 85 °C.

### 4.6. Chitosan–Genipin Hydrogel Preparation

Various chitosan–genipin gels were prepared. The gels differed between each other in terms of final volumes, chitosan concentrations and glucosamine-to-genipin molar ratios (R_D/G_). Here, it is explained how to prepare a 1.0% (m/V), R_D/G_ 90 chitosan–genipin gel (final volume = 10 mL). A total of 100 mg of hydrochloride chitosan were dissolved in 8 mL of deionized water. Then, 1 mL of 10× PBS was added in order to obtain a final 1× PBS concentration. The pH was adjusted to around 7.4 by adding aliquots of NaOH (0.1 M or 0.5 M), and deionized water was added in order to obtain a final volume of 9.8 mL. Prior to gelification, 200 μL of a 15.6 mM genipin solution, in deionized water, were mixed under magnetic stirring for about a minute. Gelification was achieved under 2 different conditions:

- On-rheometer gelation. Upon the addition of genipin, the chitosan–genipin solution was poured atop the rheometer plate. Mineral oil was used to seal the interface between the two plates in order to improve the thermal control and limit solvent evaporation. The solution was left for 3 h at 60 °C under a time sweep experiment. All the rheometer’s settings will be discussed later;
- In-well gelation. Upon the addition of genipin, the chitosan–genipin solution was poured in a 6-well plate, 2.5 mL for each well. After this, deionized water was added in the empty wells and the plate was sealed in order to prevent excessive water evaporation from the chitosan–genipin solution. The plate was then put in the oven and left for different times depending on the temperature used (2 h at 80 °C, 2.5 h at 70 °C, 3 h at 60 °C, 5 h at 45 °C, 24 h at 37 °C).

### 4.7. Rheological Measurements

Rheological measurements were performed using an HAAKE MARS III rheometer (Thermo Scientific) operating in oscillatory shear conditions. The experimental settings used to characterize chitosan gels were the following: titanium plates with 2° cone/plate geometry (Ø = 35 mm) and a gap of 0.105 mm. In the case of the on-rheometer gelation, time sweep experiments were carried out in strain-controlled conditions, with the strain, γ, of 3% kept constant throughout the experiment; frequency, ν, of 1 Hz; time of 10,800 s; T = 60 °C. The values of storage *G*′ (elastic response) and loss G″ (viscous response) moduli were recorded as a function of time. Mechanical spectra were recorded at T = 37 °C under oscillatory shear conditions, with the constant applied stress, τ, of 1 Pa (well within the linear viscoelasticity range) in the frequency range 0.01–100 Hz. At the end of the frequency sweep measurements, stress sweep experiments were performed, with ν = 1 or 0.1 Hz, respectively, stress range 1 < τ < 1000 Pa and T = 37 °C.

## Figures and Tables

**Table 1:** Acetylated chitosan samples prepared in this study.

Sample	Method	F_A_	[η] (mL/g)	$\bar{M_{w}}$ (g/mol)	M_W_ (g/mol)	$\bar{DP}$
ACh1	Homogeneous	0.44	876	280,000	199.9	1401
ACh2	Heterogeneous 60 min	0.42	392	120,000	199.8	601
ACh3	Heterogeneous 30 min	0.54	752	240,000	200.5	1197

The fraction of acetylated units, F_A_, was determined by means of ^1^H-NMR spectroscopy (see Supplementary Materials). MW stands for the molar mass of the repeating unit and was calculated on the basis of the chitosan chemical composition (note: GlcNH_2_⋅HCl was used for the calculation). Intrinsic viscosity, [*η*], values were determined at 0.1 M NaCl, 20 mM AcOH/AcONa, pH 4.5 and T = 25 °C. The weight average molar mass, $\bar{M_{w}}$, was calculated from the Mark–Houwink–Sakurada equation, $\left[\eta \right]=K\;{\bar{M_{w}}}^{a}$, with K and a as reported in [9]. $\bar{DP}$ is the average degree of polymerization, i.e., the ratio between $\bar{M_{w}}$ and MW of the chitosan repeating unit.

## Data Availability

Data availability from the corresponding author upon reasonable request.

## Supplementary Materials

The following supporting information can be downloaded at: https://www.mdpi.com/article/10.3390/gels8030194/s1, Figure S1: ^1^H-NMR spectrum; Figure S2: mechanical spectra and fitting of the experimental data. Figure S3: spectrum of the mechanical relaxation times. Figure S4: long stress sweep and fitting of the experimental data.
