# Supplementary material for: Influence of Temperature and Polymer Concentration on the Nonlinear Response of Highly Acetylated Chitosan–Genipin Hydrogels
Source: Gels. 2022 Mar 21;8(3):194. doi: 10.3390/gels8030194 (PMC8955893; doi:10.3390/gels8030194)
Supplement: Supplementary file 1 [file gels-08-00194-s001.zip › gels-1627356-supplementary.pdf]

gels

Communication

Influence of Temperature and Polymer Concentration on the Nonlinear Response of Highly Acetylated Chitosan–Genipin Hydrogels

Lorenzo Mio [1], Pasquale Sacco [1,2,3] and Ivan Donati [1,*]

[1] Department of Life Sciences, University of Trieste, Via Licio Giorgieri 5, I-34127 Trieste, Italy; lorenzo.mio@studenti.units.it
[2] AREA Science Park, Loc. Padriciano 99, I-34149 Trieste, Italy; psacco@units.it
[3] Department of Medicine, Surgery and Health Sciences, University of Trieste, Piazza dell'Ospitale 1, I-34129 Trieste, Italy
* Correspondence: idonati@units.it

Supporting Information

Determination of F_A for chitosan samples.

The degree of acetylation for chitosan samples was calculated from the ^{1}H-NMR spectrum using the integral of the signals reported in figure S1.

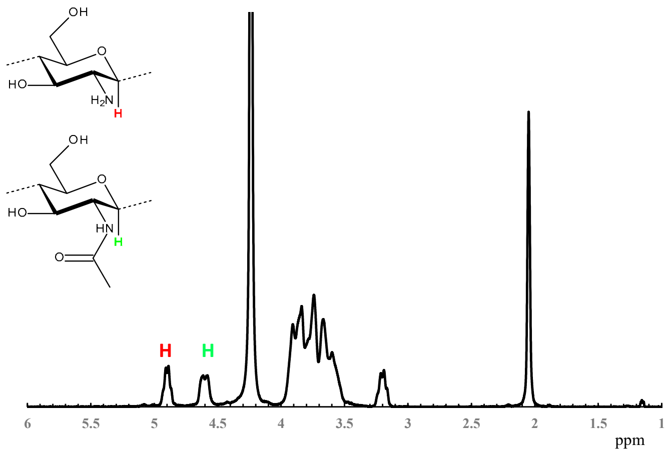

Figure S1. ^{1}H-NMR spectrum of ACh1.

The degree of acetylation, F_A, was calculated according to eq. S1:

$$F_A = \frac{I_H}{I_H + I_H} \quad \text{(S1)}$$

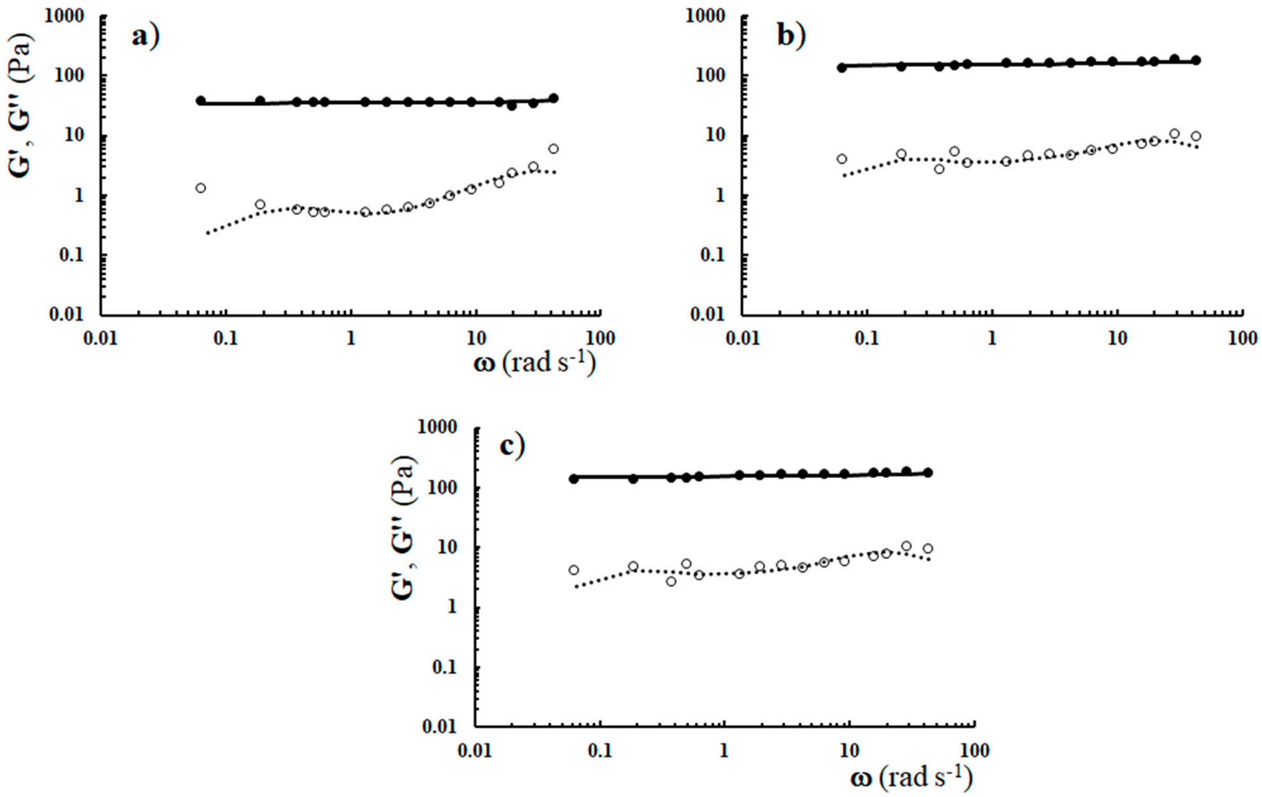

Figure S2. Dependence of G' (filled symbols) and G'' (open symbols) for ACh1 reticulated with genipin (0.31 mM). Chitosan concentration: **a**) 0.8 % (m/V); **b**) 1.0 % (m/V) and **c**) 1.3 % (m/V). Continuous line and dashed line represent the best fit of the experimental data using the generalized Maxwell model (eqs. 1 and 2).

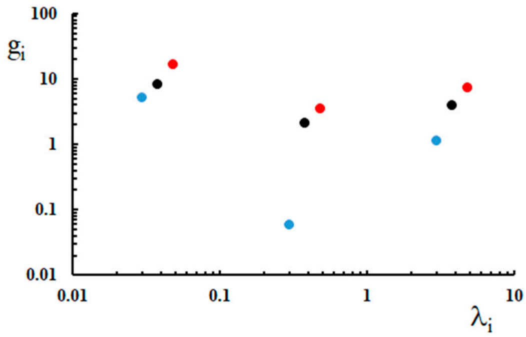

Figure S3. Dependence of g_i from λ_i obtained from the fitting of Figure S2 for ACh1 reticulated with genipin (0.31 mM). Chitosan concentration = 0.8 % (m/V) (blue); 1.0 % (m/V) (black) and 1.3 % (m/V) (red).

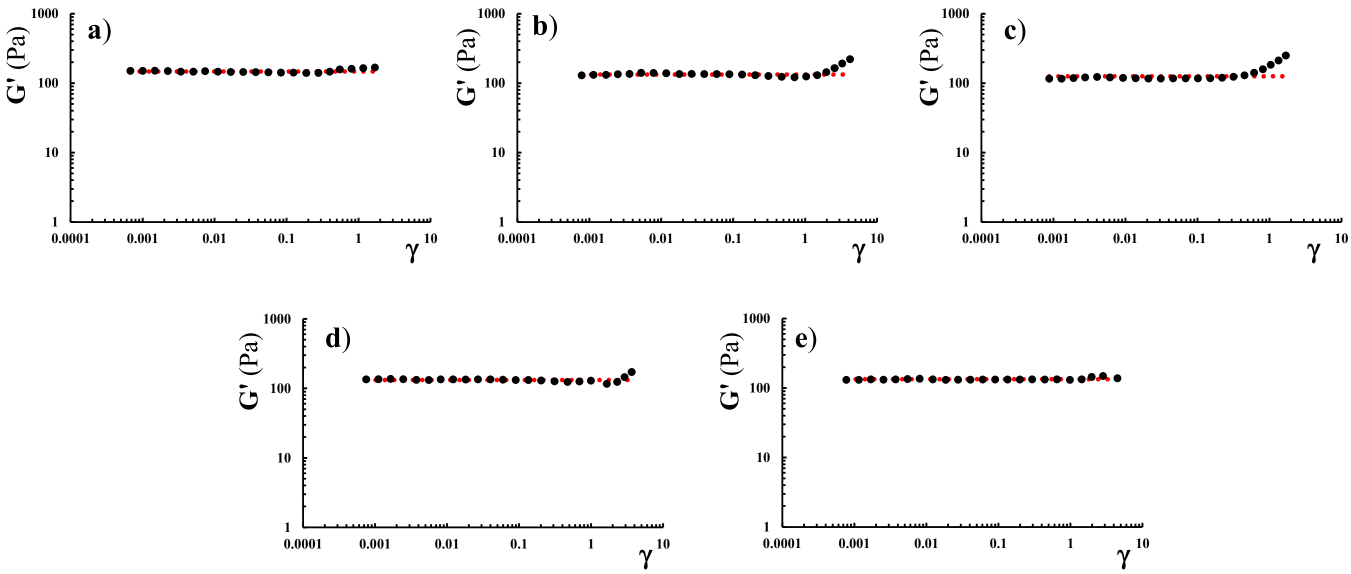

Figure S4. Stress sweep for ACh1 1% reticulated with genipin (RD/G = 90) at **a**) 37 °C, **b**) 45 °C, **c**) 60 °C, **d**) 70 °C and **e**) 80 °C. The red dotted curve is the best fit of the experimental curves using the Soskey-Winter equation (eq. 4).
